# Supplementary material for: Glycolysis inhibition induces anti-tumor central memory CD8+T cell differentiation upon combination with microwave ablation therapy
Source: Nat Commun. 2024 May 31;15:4665. doi: 10.1038/s41467-024-49059-6 (PMC11143264; doi:10.1038/s41467-024-49059-6)
Supplement: Supplementary file 1 — Supplementary Information [file 41467_2024_49059_MOESM1_ESM.pdf]

# Glycolysis inhibition induces anti-tumor central memory CD8+ T cell differentiation upon combination with microwave ablation therapy

Xinyu Tang[1,2#], Xinrui Mao[1,2#], Peiwen Ling[1,2#], Muxin Yu[1,2#], Hua Pan[3#], Jiaming Wang[1,2], Mingduo Liu[1,2], Hong Pan[1,2], Wen Qiu[4], Nan Che[5], Kai Zhang[2,6,7], Feifan Bao[8], Hongwei Peng[3], Qiang Ding[1,2], Shui Wang[1,2*], Wenbin Zhou[1,2*]

1 Department of Breast Surgery, Department of General Surgery, The First Affiliated Hospital with Nanjing Medical University, 300 Guangzhou Road, 210029, Nanjing, China;

2 Jiangsu Key Lab of Cancer Biomarkers, Prevention and Treatment, Jiangsu Collaborative Innovation Center For Cancer Personalized Medicine, School of Public Health, Nanjing Medical University, Nanjing 211166, China;

3 Department of General Surgery, Liyang Branch of Jiangsu Provincial People's Hospital, 70 Jianshe West Road, 213399, Liyang, China;

4 Department of Immunology, Nanjing Medical University, Nanjing 211166, China;

5 Department of Rheumatology and Immunology, The First Affiliated Hospital with Nanjing Medical University, 300 Guangzhou Road, 210029, Nanjing, China;

6 Pancreatic Center & Department of General Surgery, The First Affiliated Hospital with Nanjing Medical University, Nanjing 210029, Jiangsu, China;

7 Pancreas Institute of Nanjing Medical University, Nanjing 210029, Jiangsu, China;

8 The first clinical medical college of Nanjing Medical University

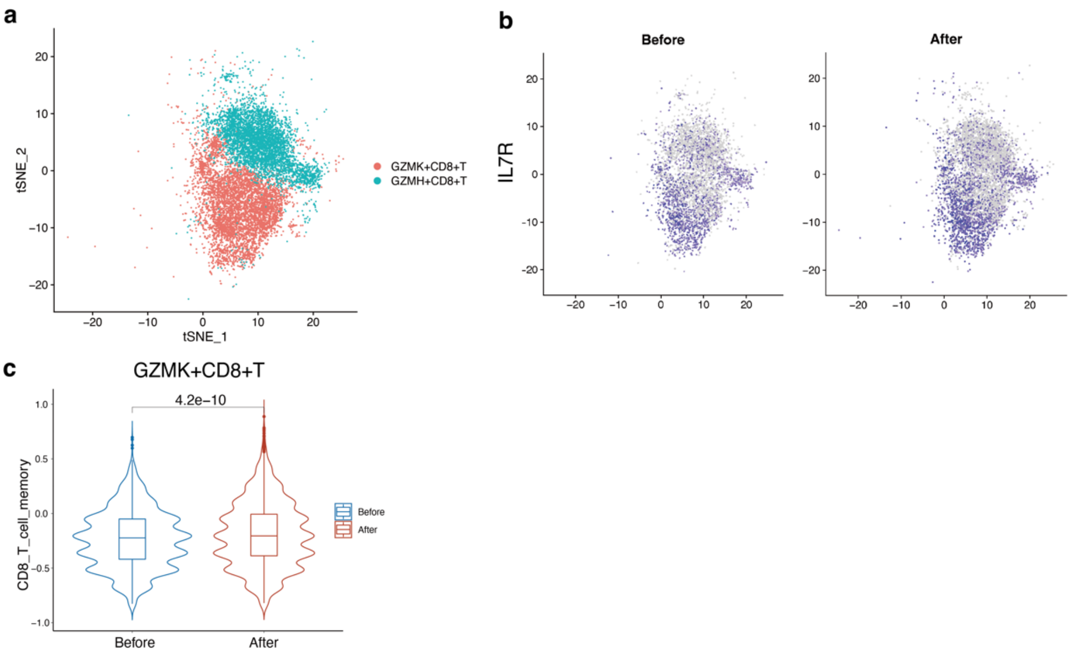


**Supplementary Fig 1. Memory and metabolic features of peripheral CD8$^+$T cells in patients with breast cancer post-local ablation, related to Fig 1. a** T-distributed stochastic neighbor embedding (t-SNE) plot of two distinct clusters demarcated by colors based on gene expression differences in CD8$^+$T cells. **b** Feature plots of *IL7R* gene expression in the CD8$^+$T cell clusters before and after MWA. **c** Violin plots of CD8+T memory score in *GZMK*$^+$CD8$^+$T cell clusters before and after MWA. Data in **(c)** was compared using the unpaired two-tailed t-test.

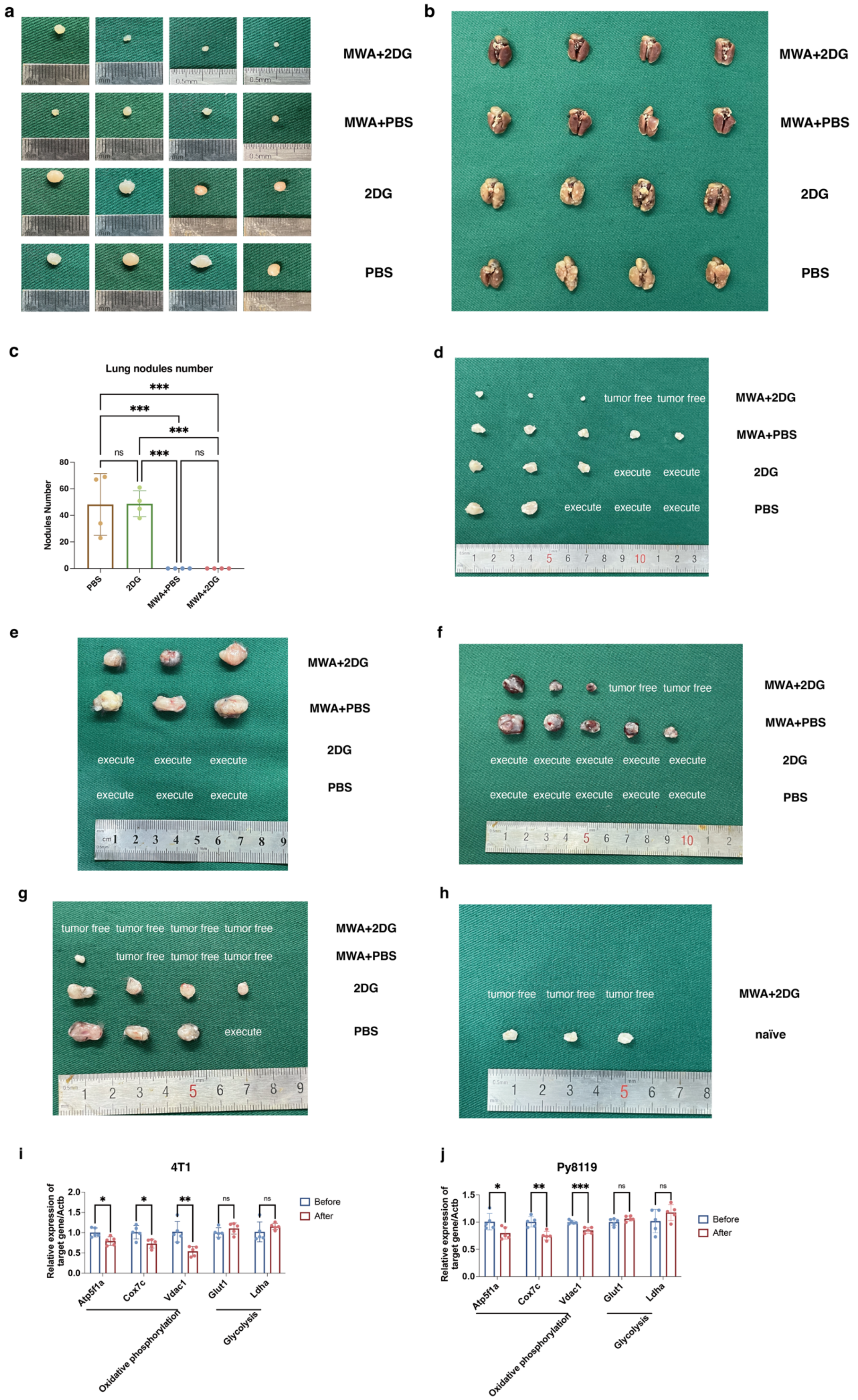

a
MWA+2DG
MWA+PBS
2DG
PBS
b
MWA+2DG
MWA+PBS
2DG
PBS
c
Lung nodules number
***
***
***
ns
***
ns
Nodules Number
0
20
40
60
80
PBS
2DG
MWA+PBS
MWA+2DG
d
tumor free tumor free
execute execute
execute execute execute
MWA+2DG
MWA+PBS
2DG
PBS
e
MWA+2DG
MWA+PBS
execute execute execute
2DG
execute execute execute
PBS
f
tumor free tumor free
MWA+2DG
MWA+PBS
execute execute execute execute execute
2DG
execute execute execute execute execute
PBS
g
tumor free tumor free tumor free tumor free
tumor free tumor free tumor free
execute
MWA+2DG
MWA+PBS
2DG
PBS
h
tumor free tumor free tumor free
MWA+2DG
naïve
i
4T1
Relative expression of target gene/Actb
0.0
0.5
1.0
1.5
2.0
*
*
**
ns
ns
Before
After
Atp5f1a
Cox7c
Vdac1
Glut1
Ldha
Oxidative phosphorylation
Glycolysis
j
Py8119
Relative expression of target gene/Actb
0.0
0.5
1.0
1.5
*
**
***
ns
ns
Before
After
Atp5f1a
Cox7c
Vdac1
Glut1
Ldha
Oxidative phosphorylation
Glycolysis

**Supplementary Fig 2 Interfering with glycolysis potentiates the anti-tumor effect of complete local ablation, related to Fig 2. a** Representative image of 4T1 TDLNs collected on day 35 (n = 4 per group). **b** Representative image of lungs harvested from 4T1 tumor-bearing mice on day 35 (n = 4 per group). **c** Number of 4T1 lung metastases in mice from the 4 groups on day 35 (n = 4 per group). **d** Representative image of re-challenged 4T1 tumors excised 15 days after the re-challenge experiment on day 35. Several mice were executed a few days after the re-challenge experiment because of tumor volume (n = 5 per group). **e** Representative image of re-challenged Py8119 tumors excised 15 days after the re-challenge experiment on day 35 (n = 3 per group). **f** Representative image of re-challenged B16F10 tumors excised 15 days after the re-challenge experiment on day 35. Several mice were executed a few days before the re-challenge experiment because of tumor volume (n = 5 per group). **g** Representative image of re-challenged MC38 tumors excised 15 days after the re-challenge experiment on day 35. One mouse was executed a few days before the re-challenge experiment because of tumor volume (n = 4 per group). **h** Representative image of re-challenged 4T1 tumors excised 15 days after the re-challenge experiment on day 140 (n = 3 per group). **i-j** Relative expression of metabolic genes of $CD8^{+}$T cells isolated from our mouse model 0 days before and 7 days after MWA (n = 5). All the experiments were repeated for at least two times. Data are mean ± SD **(c**, **i** and **j)**. Significance determined by one-way ANOVA **(c)** and two-tailed unpaired t-test **(i** and **j)**. *P < 0.05, **P < 0.01, ***P < 0.001, ****P < 0.0001. Exact p values and source data are provided as a Source Data file.

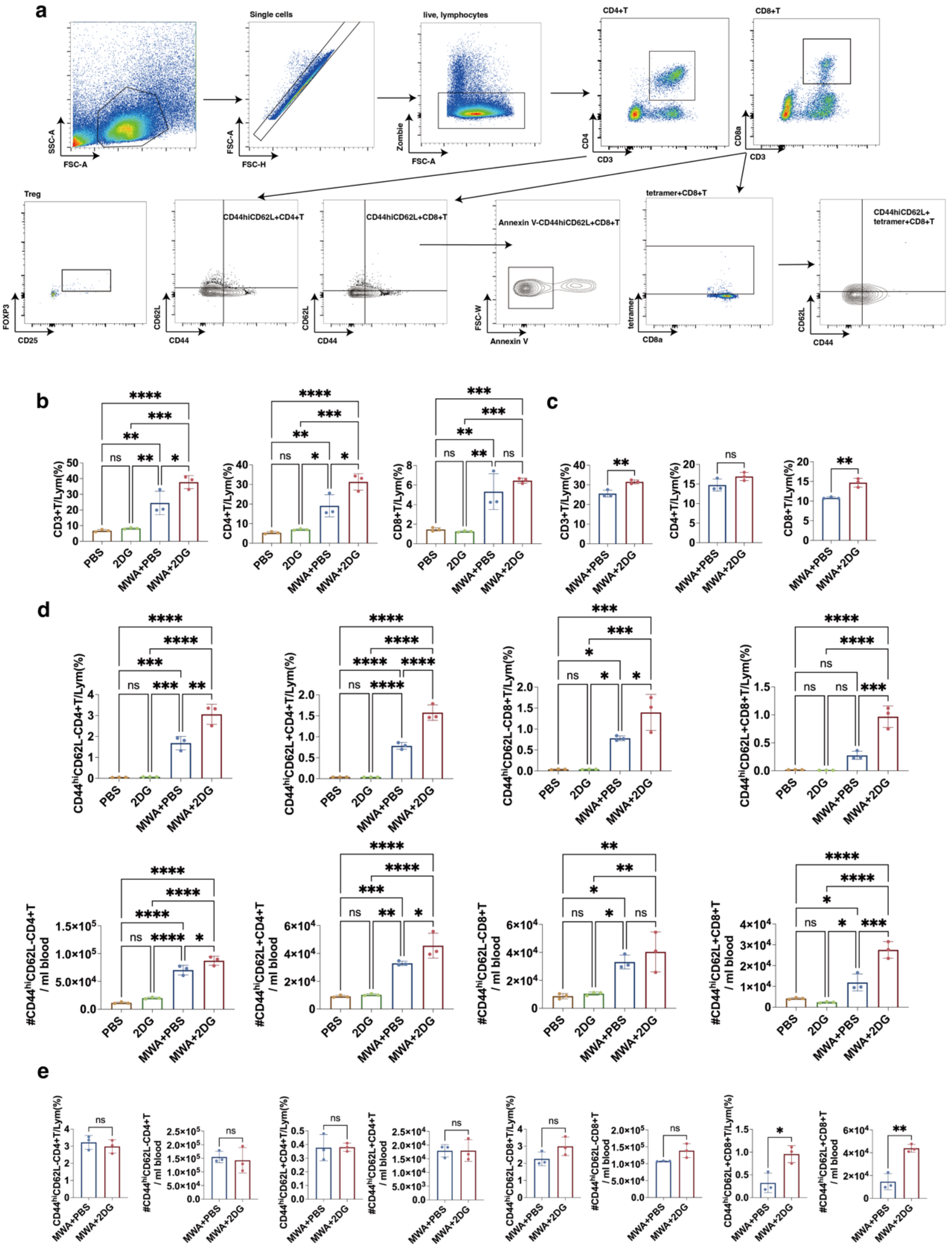


**Supplementary Fig 3 Interfering with glycolysis post-local ablation induces enhanced long-term memory phenotype of peripheral CD8$^{+}$T cells, related to Fig 3. a** Representative gating strategy for CD44$^{hi}$CD62L$^{+}$CD4$^{+}$T,

$CD44^{hi}CD62L^{+}CD8^{+}T$, $tetramer^{+}CD8^{+}T$, $CD44^{hi}CD62L^{+}tetramer^{+}CD8^{+}T$, Tregs, and non-apoptotic $CD44^{hi}CD62L^{+}CD8^{+}T$ cells. **b** and **c** Percentages of $CD3^{+}T$, $CD4^{+}T$, and $CD8^{+}T$ cells in peripheral blood of mice with 4T1 tumors (**b**) or Py8119 tumors (**c**) from the 4 groups in **(**fig 3**a)** on day 35 (n = 3 per group). **d** and **e** Percentages and absolute numbers of $CD44^{hi}CD62L^{+}CD8^{+}T$, $CD44^{hi}CD62L^{-}CD8^{+}T$, $CD44^{hi}CD62L^{+}CD4^{+}T$, and $CD44^{hi}CD62L^{-}CD4^{+}T$ cells in the peripheral blood of mice with 4T1 tumors (**d**) or Py8119 tumors (**e**) from the 4 groups in **(**fig 3**a)** on day 35 (n = 3 per group). All the experiments were repeated for at least two times. Data are mean ± SD **(b-e)**. Significance determined by one-way ANOVA **(b** and **d)** and two-tailed unpaired t-test **(c** and **e).** *P < 0.05, **P < 0.01, ***P < 0.001, ****P < 0.0001. Exact p values and source data are provided as a Source Data file.

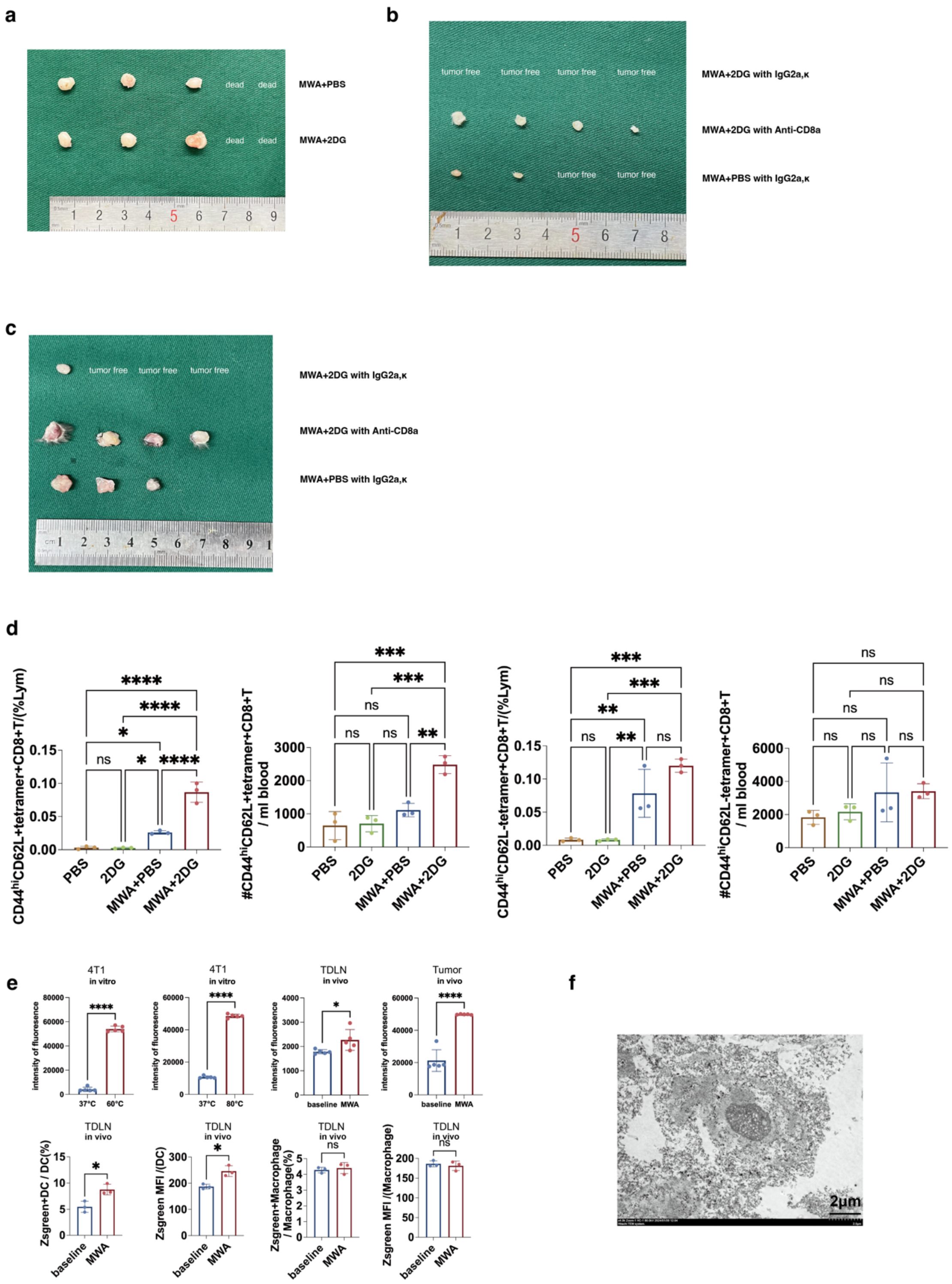


**Supplementary Fig 4 Interfering with glycolysis boosts the anti-tumor effect of local ablation in a CD8$^+$T cell-dependent manner, related to Fig 4.**

**a** Representative image of re-challenged 4T1 tumors excised 15 days after the re-challenge experiment on day 35. Several mice were dead a few days after the re-

challenge experiment (n = 5 per group). **b** Representative image of re-challenged 4T1 tumors excised 15 days after the re-challenge experiment on day 35 with or without depletion of $CD8^{+}$T cells (n = 4 per group). **c** Representative image of re-challenged Py8119 tumors excised 15 days after the re-challenge experiment on day 35 with or without depletion of $CD8^{+}$T cells (n = 4 in the two MWA+2DG groups and n = 3 in the MWA+PBS group) **d** Percentages and absolute numbers of $CD44^{hi}CD62L^{+}tetramer^{+}CD8^{+}$T and $CD44^{hi}CD62L^{-}tetramer^{+}CD8^{+}$T cells in the peripheral blood of mice with 4T1 tumors from the 4 groups in (fig 4**f**). **e** Zsgreen intensity in the supernatant liquor of 4T1-zsgreen treated with water bath (n = 5, top of fig, in vitro). Zsgreen intensity in the diluted interstitial fluid of 4T1-zsgreen primary tumor or TDLN 5h after MWA or non-treatment (n = 5, top of fig, in vivo). Percentages of zsgreen+DCs and zsgreen+macrophages and MFI of zsgreen in DCs and macrophages in the TDLNs 1 day after MWA or non-treatment (n = 3, bottom of fig). **f** Representative image of cell necrosis in the 4T1 tumor 5h after MWA by TEM. All the experiments were repeated for at least two times. Data are mean ± SD **(d-e)**. Significance determined by one-way ANOVA **(d)** and two-tailed unpaired t-test (**e**). *P < 0.05, **P < 0.01, ***P < 0.001, ****P < 0.0001. Exact p values and source data are provided as a Source Data file.

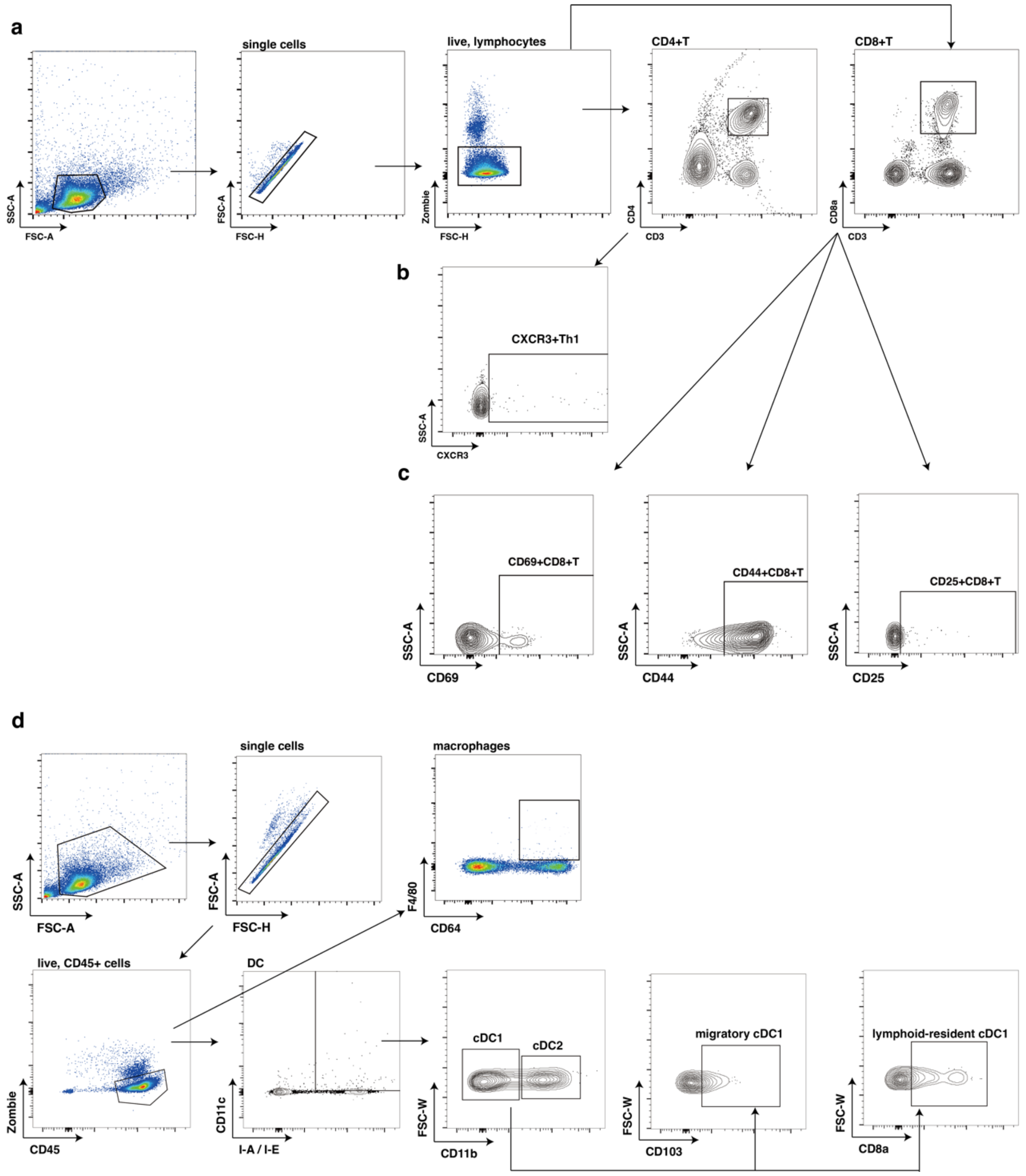


**Supplementary Fig 5 Immune microenvironment in TDLN is more activated by the combination therapy compared with local ablation alone, related to Fig 5. a-d** Representative gating strategy for Th1, macrophages, $CD69^{+}CD8^{+}T$, $CD44^{+}CD8^{+}T$, $CD25^{+}CD8^{+}T$, DC, cDC1, cDC2, migratory cDC1, and resident cDC1 cells in TDLNs of mice.

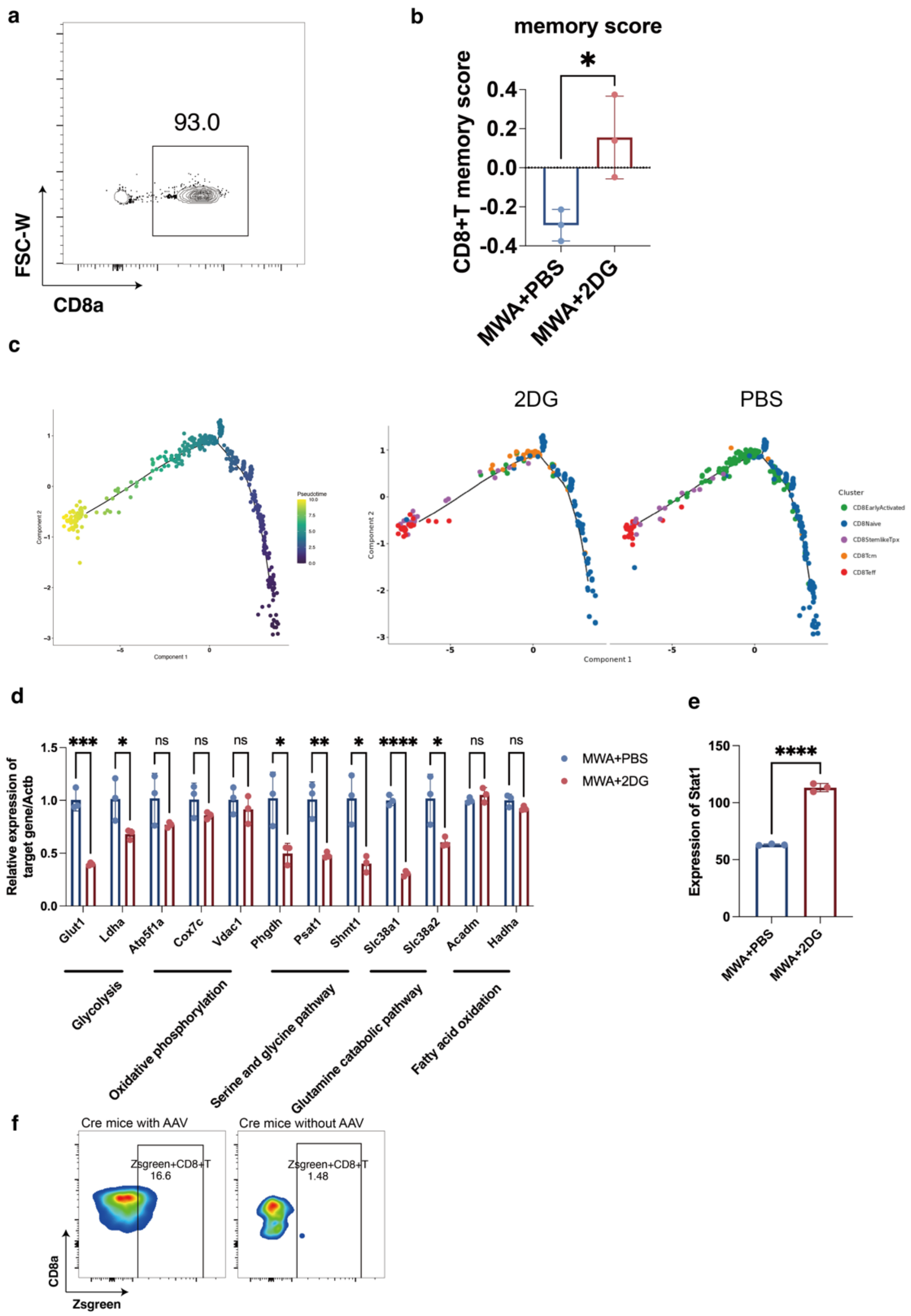


**Supplementary Fig 6 Enhancement of CD8$^{+}$T$_{CM}$ cell differentiation after the combination therapy does not occur in TDLN but mainly in peripheral blood, related to Fig 6. a** Validation of the sorting of CD8$^{+}$T cells. **b** Memory scores of CD8+T cells isolated from spleens of mice in the MWA+PBS and MWA+2DG groups. **c** Pseudo-time trajectory showing the distribution of tumor-specific CD8+T

cells from peripheral blood of mice in the MWA+PBS and MWA+2DG groups (n = 2). **d** Relative expression of metabolic genes in CD8+T cells isolated from the spleens of mice on day 15 in the MWA+PBS and MWA+2DG groups (n = 3). **e** Expression of *Stat1* in the RNA-seq of CD8+T cells from the spleens of mice in the MWA+PBS and MWA+2DG groups (n = 3). **f** The transduction efficiency of AAV in CD8a-Cre mice. The experiments were repeated for at least two times (**a**, **d,** and **f**). Data are mean ± SD **(b**, **d**, and **e)**. Significance determined by two-tailed unpaired t-test **(b**, **d**, and **e).** *P < 0.05, **P < 0.01, ***P < 0.001, ****P < 0.0001. Exact p values and source data are provided as a Source Data file.

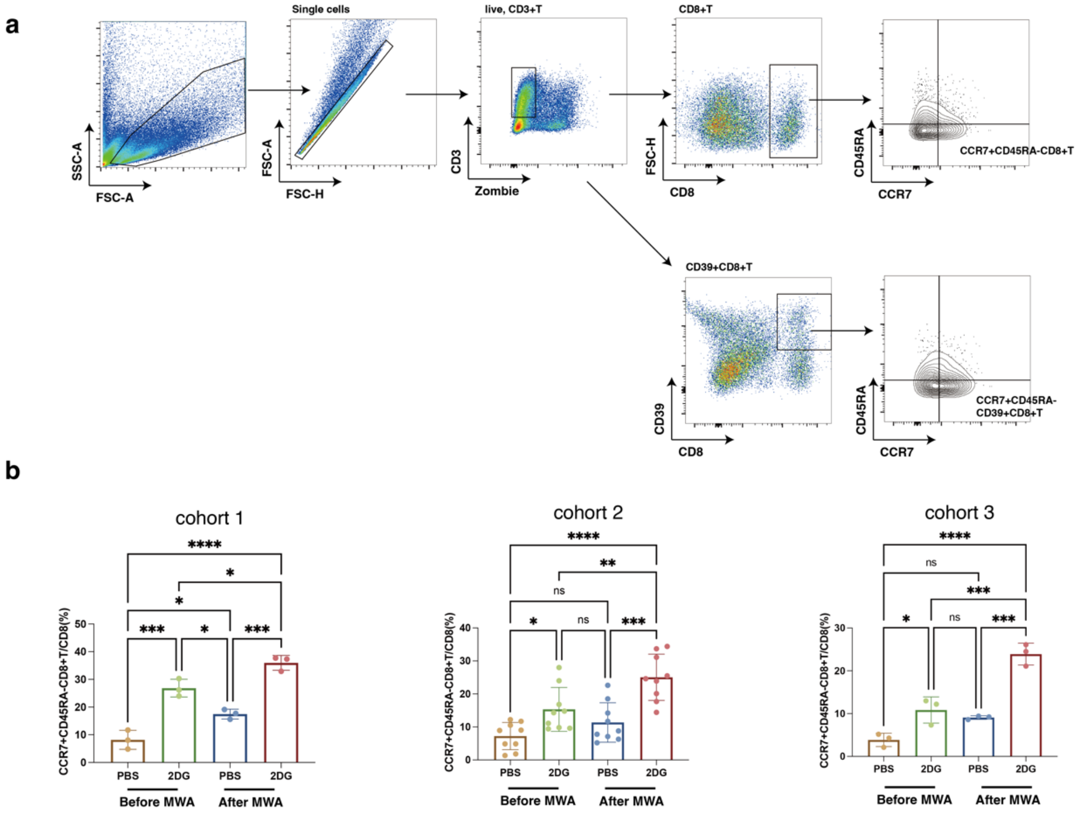


**Supplementary Fig 7 Peripheral CD8$^{+}$T cells of patients post MWA of local tumors differentiate into CD8$^{+}$T$_{CM}$ cells by inhibiting glycolysis in vitro, related to Fig 7.**

**a** Representative gating strategy for CD39$^{+}$CD8$^{+}$T$_{CM}$ and CD8$^{+}$T$_{CM}$ cells from PBMCs of patients with breast cancer or liver tumors. **b** Percentages of CD8$^{+}$T$_{CM}$ cells in the 4 groups of PBMCs. Data are mean ± SD **(b)**. Significance determined by one-way ANOVA **(b).** *P < 0.05, **P < 0.01, ***P < 0.001, ****P < 0.0001. Exact p values and source data are provided as a Source Data file.

**Supplementary Table 1**. Characteristics of enrolled patients for microwave ablation

| Variables | Cohort 1 | Cohort2 | Cohort3 |
|---|---|---|---|
| Age (mean, range), year | 76.3(71-84) | 53.4(39-69) | 60(52-67) |
| Tumor size (mean, range), mm | 20(15-22) | 18(10-30) | 28(11-60) |
| Hormone receptor positive | 2/3 | 5/9 | - |
| Her 2 positive | 1/3 | 1/9 | - |
| Complete ablation | 3/3 | 9/9 | 3/3 |

**Supplementary Table 2**. The primer sequences of qRT-PCR

| Gene | Sequence (5' to 3') Forward | Sequence (5' to 3') Reverse |
|---|---|---|
| *Shmt1* | CAGGGCTCTGTCTGATGCAC | CGTAACGCGCTCTTGTCAC |
| *Actb* | GGCTGTATTCCCCTCCATCG | CCAGTTGGTAACAATGCCATGT |
| *Atp5f1a* | TCTCCATGCCTCTAACACTCG | CCAGGTCAACAGACGTGTCAG |
| *Phgdh* | ATGGCCTTCGCAAATCTGC | AGTTCAGCTATCAGCTCCTCC |
| *Psat1* | CAGTGGAGCGCCAGAATAGAA | CCTGTGCCCCTTCAAGGAG |
| *Cox7c* | ATGTTGGGCCAGAGTATCCG | ACCCAGATCCAAAGTACACGG |
| *Vdac1* | CCCACATACGCCGATCTTGG | GTGGTTTCCGTGTTGGCAGA |
| *Glut1* | CAGTTCGGCTATAACACTGGTG | GCCCCCGACAGAGAAGATG |
| *Ldha* | TGTCTCCAGCAAAGACTACTGT | GACTGTACTTGACAATGTTGGGA |
| *Slc38a1* | AGCAACGACTCTAATGACTTCAC | CCTCCTACTCTCCCGATCTGA |
| *Slc38a2* | TAATCTGAGCAATGCGATTGTGG | AGATGGACGGAGTATAGCGAAAA |
| *Acadm* | AGGGTTTAGTTTTGAGTTGACGG | CCCCGCTTTTGTCATATTCCG |
| *Hadha* | TGCATTTGCCGCAGCTTTAC | GTTGGCCCAGATTTCGTTCA |
| Sh*Stat1*-1 | TGCATTTGCCGCAGCTTTAC | GTTGGCCCAGATTTCGTTCA |
| Sh*Stat1*-2 | TGCATTTGCCGCAGCTTTAC | GTTGGCCCAGATTTCGTTCA |

**Supplementary Table 3**. The sequences of shRNA

| Gene | Sequence |
|---|---|
| Sh*Stat1*-1 | CCGAAGAACTTCACTCTCTTA |
| Sh*Stat1*-2 | CGCCTTTGGGAAGTATTATT |
